# Determinants of parliamentary health funding allocation in Brazil: A national cross-sectional study

**DOI:** 10.1016/j.clinsp.2026.101198

**Published:** 2026-10-14

**Authors:** Mariana G. Magon, Marcio R. Facanali, José Maria Soares, Sarah Emmely Fazanaro, Everson L.A. Artifon

**Affiliations:** a Department of Surgery, Faculdade de Medicina da Universidade de São Paulo, São Paulo, SP, Brazil; b Department of Gastroenterology, Faculdade de Medicina da Universidade de São Paulo, São Paulo, SP, Brazil; c Department of Gynecology and Obstetrics, Faculdade de Medicina da Universidade de São Paulo, São Paulo, SP, Brazil

**Keywords:** Health policy, Resource allocation, Health governance, Health services research, Resource mobilization, Brazil

## Abstract

Parliamentary amendments are an important mechanism for health resource allocation in Brazil, but little is known about the characteristics of actors involved in mobilizing these resources. This national cross-sectional web-based survey included 276 respondents, each reporting on the usual amendment allocation pattern of a parliamentary office in 2025. The outcome was categorized as higher-magnitude (> BRL 1,000,000; n = 124) or lower-magnitude (≤ BRL 1,000,000; n = 152). Associations with individual, institutional, political, and allocation-related variables were examined using multivariable logistic regression with backward selection based on the Akaike information criterion. Higher-magnitude allocations were associated with relevance attributed to party affiliation (adjusted Odds Ratio [aOR = 2.80], 95% Confidence Interval [95% CI 1.46–5.55]), prioritization of capital investment (aOR = 1.79, 95% CI 1.07–3.02), and representation by a professional advisory intermediary (aOR = 1.94, 95% CI 1.16–3.29). Extroverted personality profile (aOR = 0.36, 95% CI 0.15–0.81), an advisory position within the parliamentary office (aOR = 0.51, 95% CI 0.28–0.92), and prioritization of higher-complexity care (aOR = 0.58, 95% CI 0.34–0.97) were inversely associated with higher-magnitude allocation. The final model showed moderate apparent discrimination (AUC = 0.716). Reported amendment magnitude was associated with political alignment, allocation strategy, and intermediary characteristics. These exploratory findings should be interpreted in light of the cross-sectional design, purposive sampling, and respondent-reported outcome.

## Highlights

• Political alignment was associated with higher-magnitude health funding.
• Capital investment priority was associated with higher-magnitude allocation.
• Professional intermediaries were associated with higher-magnitude allocations.
• The final model showed moderate apparent discrimination (AUC = 0.716).

## Introduction

The governance of health resource allocation represents a central challenge for health systems worldwide, particularly in decentralized contexts where discretionary funding mechanisms coexist with formal budgetary structures. While discretionary funding may enhance responsiveness to local needs, it also introduces substantial variation in allocation patterns, equity, and performance across health systems.^1^

The health policy literature has extensively examined institutional and structural determinants of resource allocation. Studies have highlighted the influence of fiscal decentralization, governance arrangements, political alignment between levels of government, and regulatory capacity on health spending patterns and outcomes^2^^,^^3^ From a political economy perspective, legislative bargaining, agenda-setting dynamics, and policy networks have been shown to affect both the volume and destination of public resources, including health expenditures^4^ Together, these approaches have substantially advanced understanding of *where* and *why* health resources are allocated.

However, despite this extensive body of work, empirical research has largely overlooked the individual-level actors who operationalize institutional and political processes related to health funding. Recent conceptual contributions have begun to frame health resource mobilization as a specialized professional activity, emphasizing its strategic, technical, and relational dimensions^5^ These discussions highlight the growing relevance of healthcare resource mobilizers and intermediary actors in health systems where institutional budgets are insufficient to meet expanding demands^6^ Nevertheless, existing approaches remain predominantly descriptive and context-specific, providing limited empirical evidence on how individual-level characteristics of these actors are independently associated with the magnitude and patterns of public health resource mobilization.

This evidence gap may limit the ability of health institutions and policymakers to adopt data-informed criteria for selecting, training, and organizing professionals responsible for resource acquisition.

Addressing this gap is particularly relevant in contexts where discretionary health funding represents a substantial share of public investment and where competition for resources is intense. Understanding which attributes of intermediaries are associated with higher-magnitude discretionary allocation patterns may contribute not only to improved funding outcomes but also to enhanced transparency, accountability, and institutional performance. From a policy perspective, shifting the analytical focus from institutions alone to the agents who operationalize funding decisions offers a complementary and necessary dimension to contemporary health systems research.

In this context, the present study aims to characterize the profile of healthcare resource mobilizers and examine individual-level determinants associated with parliamentary amendment allocation patterns in Brazil. By providing empirical evidence on intermediary-level factors associated with discretionary health funding, this study may inform institutional governance and capacity-building strategies in resource-limited health systems. Although grounded in the Brazilian discretionary funding framework, the analytical approach adopted here may inform understanding of similar allocation dynamics in other decentralized health systems.

## Materials and methods

### Study design

We conducted a national cross-sectional web-based survey in 2025 to examine individual-level determinants associated with the magnitude of discretionary public health funding mobilized by parliamentary offices.

### Setting and participants

Each questionnaire was completed once per parliamentary office by a respondent directly involved in amendment-related processes. All respondents provided information referring to a specific parliamentary office with which they were formally affiliated. Responses referred to the usual allocation patterns of each parliamentary office rather than to a single amendment event. Therefore, the unit of analysis was the parliamentary office, and outcomes should be interpreted as respondent-reported allocation patterns rather than audited administrative records. Participants were purposively sampled from parliamentary offices engaged in discretionary health resource mobilization at federal and state levels in Brazil. Eligible respondents included parliamentary advisors, professional intermediaries formally linked to the office, and direct beneficiary representatives acting within the office’s amendment processes.

Invitations were distributed electronically via institutional email using a structured questionnaire hosted on Google Forms (Google LLC). Participant recruitment was conducted between August 17, 2025 and November 18, 2025. Participation was voluntary and anonymous. No personally identifiable information was collected. Completion of the survey was considered provision of informed consent.

### Survey instrument

The questionnaire was developed based on prior conceptual and empirical work on discretionary funding mechanisms and public health resource mobilization. It consisted of structured, closed-ended questions organized into four conceptual domains: 1) Sociodemographic and professional characteristics; 2) Behavioral and interpersonal attributes; 3) Political-institutional relevance criteria; 4) Allocation decision preferences.

The instrument was designed to capture both structural and individual-level determinants potentially associated with differential funding outcomes.

The questionnaire was specifically developed by the investigators for the purposes of this study based on prior conceptual and empirical literature on health resource mobilization and discretionary funding mechanisms.

### Outcome definition

The primary outcome was the office-level pattern of amendment magnitude in discretionary public health funding. Respondents were asked to indicate the typical value range of parliamentary amendments mobilized by their respective office.

In the original dataset, amendment values were categorized in Brazilian Reais (BRL), with a threshold of BRL 1,000,000 distinguishing higher-magnitude from lower-magnitude allocations. Monetary values are reported in Brazilian Reais (BRL). The primary outcome was therefore modeled as a binary variable reflecting whether the respondent’s office typically mobilizes:

• Higher-magnitude amendments (> BRL 1,000,000);
• Lower-magnitude amendments (≤ BRL 1,000,000).

This cutoff represents a structurally meaningful distinction in funding scale within the Brazilian discretionary allocation framework and captures differences in reported allocation patterns across parliamentary offices.

### Independent variables

Independent variables were grouped into theoretically informed domains:

1. Individual Characteristics of the Resource Mobilizer:

• Sex;
• Age (≥ 35 vs. < 35-years).
• Personality profile (extroverted vs. introverted).
• Type of representation (professional advisory intermediary vs. direct beneficiary representative).
• Professional presentation.

2. Institutional Positioning:

• Position within the parliamentary office.
• Educational level.

3. Political-strategic criteria:

• Relevance attributed to political articulation.
• Relevance attributed to technical expertise.
• Relevance attributed to party affiliation.
• Political alignment between mobilizer and beneficiary.
• Relevance attributed to religion.

4. Allocation Preferences:

• Current expenditure vs. capital investment.
• Prioritization of primary vs. secondary/tertiary care.
• Type of funded object (equipment, services, infrastructure).
• Degree of project selection autonomy.
• Transfer mechanism.

In this study, “professional advisory intermediary” refers to an external specialized agent who operates as a broker between parliamentary offices and beneficiary institutions, whereas “advisory position within the parliamentary office” refers to staff members formally embedded within the parliamentary office structure.

Personality profile was assessed through a single-item forced-choice question in which respondents classified the primary resource mobilizer associated with the office as predominantly extroverted or introverted. This measure reflects perceived behavioral style rather than a validated psychometric assessment and should be interpreted as an exploratory proxy of interpersonal orientation. Variables referring to relevance criteria were originally measured using ordinal response categories and were dichotomized for regression analysis based on theoretically informed thresholds.

### Statistical analysis

All statistical analyses were performed using RStudio® version 2024.04.2 + 764.

Categorical variables were described using absolute and relative frequencies. Associations between independent variables and amendment value category were assessed using Pearson’s Chi-Squared test.

Univariable logistic regression models were constructed to estimate crude associations between each predictor and the likelihood of higher-magnitude amendment allocation. Variables with p < 0.20 in univariable analysis were included in a multivariable logistic regression model.

Model refinement was performed using backward stepwise selection based on the Akaike Information Criterion (AIC).

Model adequacy was assessed through:

• Variance Inflation Factor (VIF < 5) to evaluate multicollinearity.
• Cook’s distance (< 1) to detect influential observations.
• Area Under the Receiver Operating Characteristic Curve (AUC) to assess discrimination.

Adjusted associations are presented as Odds Ratios (OR) with 95% Confidence Intervals (95% CI). Statistical significance was defined as a two-sided p-value < 0.05.

### Model assessment

The full candidate model and the final parsimonious model were compared using the Akaike Information Criterion (AIC), Bayesian Information Criterion (BIC), and Area Under the receiver operating Characteristic Curve (AUC). Multicollinearity and influential observations were assessed using variance inflation factors and Cook’s distance, respectively.

### Ethical considerations

This study was approved by the Research Ethics Committee of the Faculdade de Medicina da Universidade de São Paulo (CAPPesq), under protocol number 8.151.946 (CAAE 75513123.4.0000.0068).

The study was conducted in accordance with Brazilian National Health Council Resolution n° 466/2012. It was based on an anonymous, web-based survey, and no identifiable personal data were collected.

Participation was voluntary, and electronic informed consent was obtained from all participants prior to survey completion.

## Results

### Sample characteristics and profile of healthcare resource mobilizers

A total of 276 respondents were included in the final analysis. Most participants held advisory roles within parliamentary offices (74.3%), and the majority had completed higher education or postgraduate training (84.1%). Respondents represented 23 Brazilian federative units, with the largest proportions from the Federal District (57.6%) and São Paulo (18.5%), and were predominantly female (61.6%). The profile of resource mobilizers described by respondents was predominantly male (79.0%), aged ≥ 35-years (83.3%), with extroverted personality being the most frequently reported profile (88.4%). Professional presentation was reported as appropriate in 97.1% of cases, and representation was balanced between professional advisory intermediaries (54.0%) and direct beneficiary representatives (46.0%). Overall, 152 offices (55.1%) were classified in the lower-magnitude group and 124 (44.9%) in the higher-magnitude group. Full sample characteristics are summarized in Table 1.

**Table 1:** Characteristics of respondents and resource mobilizers (n = 276).

Characteristic	n (%)
**Respondent profile**	
Female	170 (61.6)
Male	103 (37.3)
Sex not reported	3 (1.1)
Higher education completed	128 (46.4)
Postgraduate training	104 (37.7)
Advisory position in parliamentary office	205 (74.3)
**Resource mobilizer profile**	
Male	218 (79.0)
Age ≥35-years	230 (83.3)
Extroverted personality	244 (88.4)
Professional advisory intermediary	149 (54.0)
Direct beneficiary representative	127 (46.0)
Appropriate professional presentation	268 (97.1)
**Amendment magnitude**	
≤ BRL 1,000,000	152 (55.1)
> BRL 1,000,000	124 (44.9)

Values are presented as n (%). Percentages are calculated based on the total sample (n = 276). Minor discrepancies may reflect rounding. Missing responses are explicitly indicated.

### Independent determinants of higher-magnitude amendment patterns

In multivariable logistic regression, six variables remained independently associated with offices that typically mobilize higher-magnitude amendments (defined as > BRL 1,000,000). The largest adjusted association was observed for relevance attributed to party affiliation (adjusted OR = 2.80; 95% CI 1.46–5.55; p = 0.002). Prioritization of capital investment (adjusted OR = 1.79; 95% CI 1.07–3.02; p = 0.028) and representation by a professional advisory intermediary (adjusted OR = 1.94; 95% CI 1.16–3.29; p = 0.013) were also positively associated with belonging to the higher-magnitude amendment group.

Conversely, an extroverted personality profile (adjusted OR = 0.36; 95% CI 0.15–0.81; p = 0.015), holding an advisory position within the parliamentary office (adjusted OR = 0.51; 95% CI 0.28–0.92; p = 0.026), and prioritization of higher-complexity care levels (adjusted OR = 0.58; 95% CI 0.34–0.97; p = 0.040) were independently associated with lower odds of belonging to the higher-magnitude amendment group. Adjusted estimates are presented in Table 2.

**Table 2:** Factors associated with higher-magnitude parliamentary amendment allocation patterns (multivariable logistic regression, n = 276).

Predictor	Adjusted OR	95% CI	p-value
Relevance attributed to party affiliation	2.80	1.46–5.55	0.002
Prioritization of capital investment	1.79	1.07–3.02	0.028
Representation by professional advisory intermediary	1.94	1.16–3.29	0.013
Extroverted personality profile	0.36	0.15–0.81	0.015
Advisory position within parliamentary office	0.51	0.28–0.92	0.026
Prioritization of higher-complexity care	0.58	0.34–0.97	0.040

OR, Odds Ratio; CI, Confidence Interval. Outcome defined as offices that typically mobilize higher-magnitude amendments (> BRL 1,000,000). Model derived using backward stepwise selection based on Akaike Information Criterion (AIC). Area under the ROC curve (AUC) = 0.716. Reference categories: no relevance attributed to party affiliation; current expenditure; direct beneficiary representative; introverted profile; no advisory position; and primary care.

### Allocation patterns by amendment value category

Bivariate comparisons showed that certain decision criteria and allocation preferences differed meaningfully between lower-magnitude and higher-magnitude amendments (Table 3).

**Table 3:** Selected bivariate comparisons by usual amendment magnitude category (office level).

Variable	Lower-magnitude (n = 152)	Higher-magnitude (n = 124)	p-value
Relevance attributed to party affiliation	17 (11.2)	35 (28.2)	<0.001
Capital investment prioritized	48 (31.6)	58 (46.8)	0.014
Extroverted personality profile	141 (92.8)	103 (83.1)	0.021

Values are n (column %) within each amendment-magnitude category. Higher-magnitude amendments were defined as > BRL 1,000,000. p-values were derived from Pearson’s Chi-Squared test.

Relevance attributed to party affiliation was more frequently reported among higher-magnitude amendments than lower-magnitude amendments (28.2%vs. 11.2%; p < 0.001). Capital investment was also more common among higher-magnitude amendments (46.8%vs. 31.6%; p = 0.014). In addition, extroverted personality predominated in both groups but was proportionally less frequent in the higher-magnitude category (83.1%vs. 92.8%; p = 0.021). This pattern was consistent with multivariable findings.

### Conceptual synthesis and model performance

Taken together, the results support a conceptual structure in which higher-magnitude allocation is associated with three interacting axes: political alignment (particularly relevance attributed to party affiliation), allocation strategy (investment-oriented preferences), and intermediary profile (representation structure and behavioral attributes). This proposed framework is summarized in Fig. 1.

**Fig. 1:**
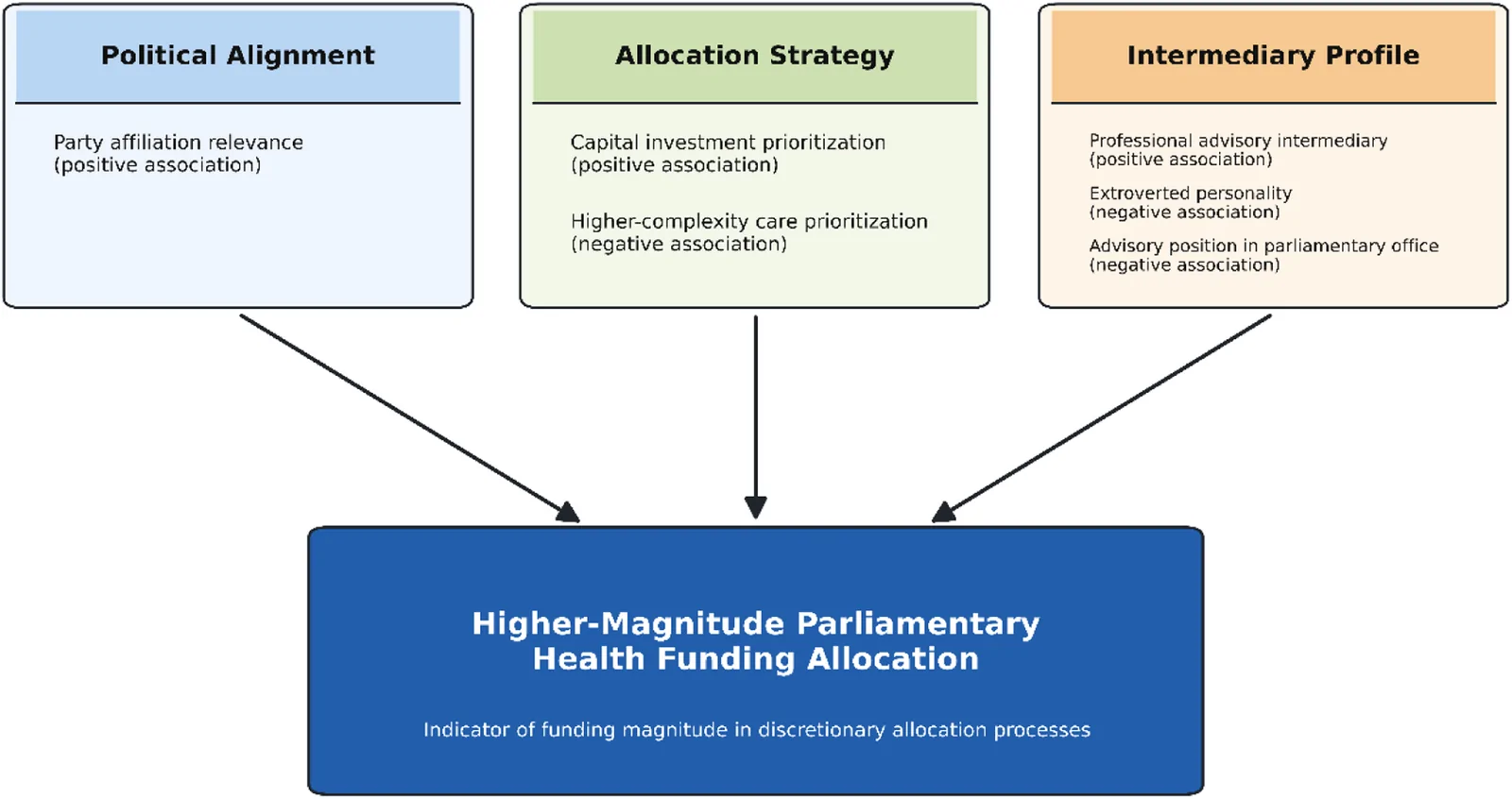
Conceptual framework of determinants associated with higher-magnitude parliamentary health funding allocation in Brazil. The framework summarizes three domains associated with allocation magnitude: political alignment, allocation strategy, and intermediary profile. Positive and negative associations indicate the direction of the relationship with higher-magnitude allocations.

Compared with the full candidate model, the final parsimonious model had lower AIC (356.97 vs. 364.17) and BIC (382.31 vs. 407.61), with similar apparent discrimination (AUC 0.716 vs. 0.727), as shown in Fig. 2.

**Fig. 2:**
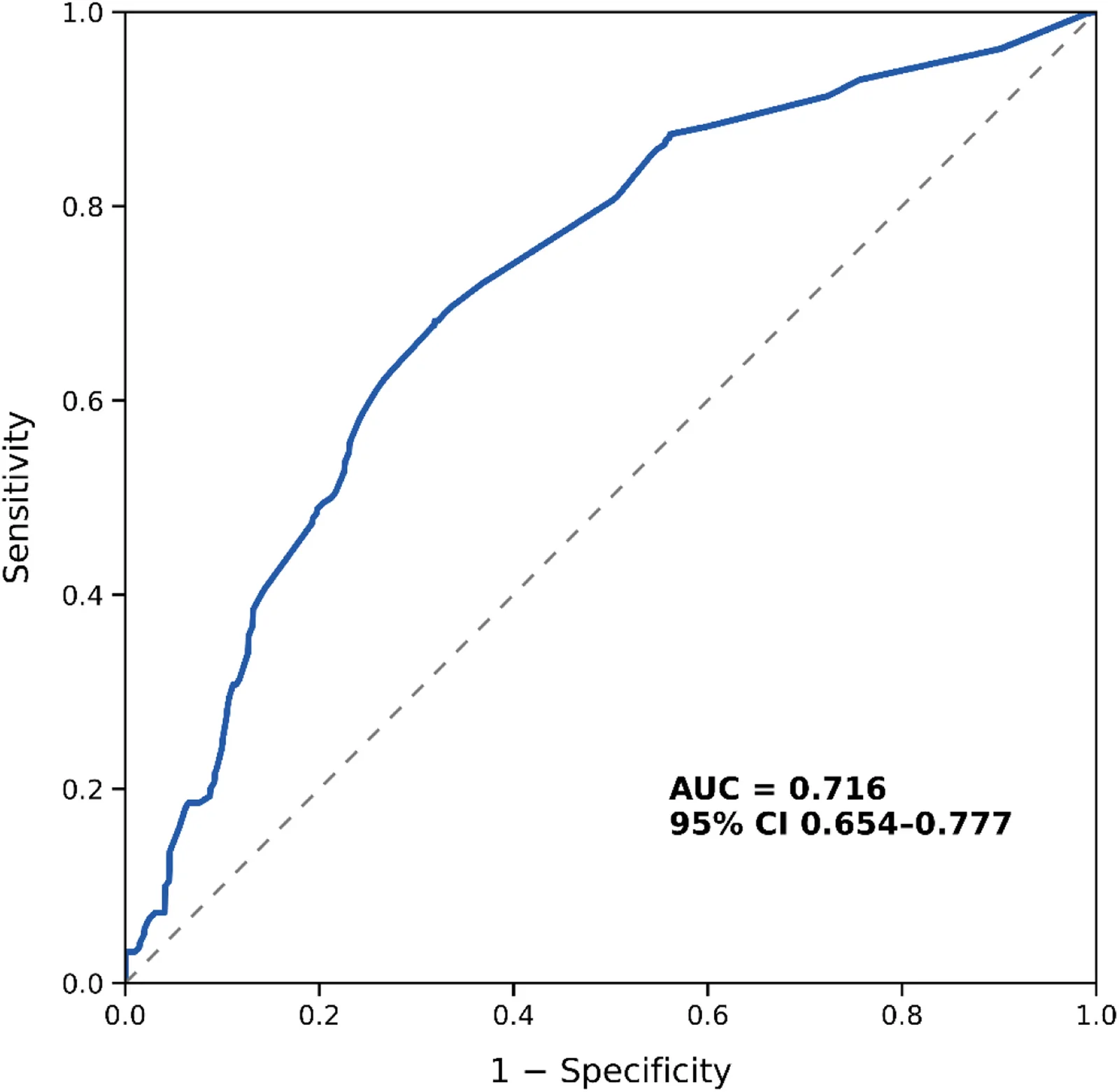
Receiver operating characteristic curve of the final parsimonious multivariable model predicting higher-magnitude parliamentary health funding allocation. The solid blue line represents the model’s discriminatory performance, whereas the dashed diagonal line represents discrimination equivalent to chance. The model achieved an area under the curve of 0.716 (95% CI 0.654–0.777).

## Discussion

This study characterized the profile of healthcare resource mobilizers and examined individual-level determinants associated with higher-magnitude parliamentary amendment allocation patterns. Three main domains emerged from the analysis: political alignment, allocation strategy, and intermediary profile. By shifting analytical focus from institutional architecture to the actors operating within discretionary funding systems, our findings contribute to an underexplored dimension of health resource governance.

### Political alignment and the political economy of allocation

The largest adjusted association was observed for relevance attributed to party affiliation. This finding aligns with political economy literature demonstrating that discretionary public spending is often shaped by partisan alignment and legislative bargaining dynamics.^2^, ^3^, ^4^ While prior studies have emphasized institutional arrangements, intergovernmental fiscal transfers, and agenda-setting mechanisms, fewer have examined how these macro-structures are operationalized at the level of individual decision-making actors.^4^^,^^7^

Our findings suggest that political alignment is not merely a structural background condition but an actively mobilized criterion in higher-magnitude allocation processes. This reinforces theoretical models positing that distributive politics operates through relational and network-based mechanisms rather than purely technocratic criteria. Importantly, technical expertise was almost universally valued across amendment categories, yet it did not independently differentiate higher-magnitude allocation. This indicates that technical legitimacy may function as a baseline requirement, while political alignment becomes decisive when allocation stakes increase.

These findings can also be interpreted through a principal–agent lens. Parliamentary offices (principals) delegate negotiation and mobilization functions to intermediaries (agents). Their individual attributes and strategic orientations may affect the scale of resources secured. In this sense, allocation magnitude may partly reflect agent-level variation within a common institutional structure.

### Allocation strategy and investment logic

Capital investment prioritization independently was associated with higher-magnitude amendments, suggesting a structural shift in expenditure logic as funding magnitude increases. Larger amendments appear more frequently directed toward investment rather than recurrent expenditure.

This pattern may reflect both political visibility incentives and strategic signaling mechanisms. Infrastructure and capital expenditures are often more politically salient and allow clearer attribution of credit^2^ From a governance perspective, such allocation behavior may simultaneously reflect strategic positioning and risk management within discretionary funding environments.

The inverse association between prioritization of higher-complexity care and higher-magnitude amendments further underscores the political calculus underlying allocation decisions. High-complexity services may be less geographically visible or politically attributable than capital investments in primary care infrastructure. These dynamics resonate with broader analysis of distributive politics and fiscal federalism in decentralized health systems . From a fiscal federalism perspective, discretionary health funding operates within a framework of vertical and horizontal intergovernmental bargaining, where allocation decisions reflect both formal transfer rules and informal political negotiation. Our findings suggest that individual-level actors may influence how this “decision space” is exercised in practice, shaping the effective translation of fiscal authority into large-scale allocation outcomes.

### Intermediary profile and the human factor in resource mobilization

A relevant contribution of this study is the identification of intermediary-level determinants. Representation by professional advisory intermediaries was positively associated with higher-magnitude amendments, whereas an extroverted personality profile and holding an advisory position within the parliamentary office were negatively associated with higher-magnitude allocation. The divergent effects observed for external professional intermediaries and internal advisory staff suggest that institutional positioning matters: externally positioned brokers may operate with greater strategic flexibility and network reach in higher-magnitude negotiations, whereas internally embedded advisors may face greater procedural constraints within parliamentary office routines.

The positive association of professional intermediaries suggests that specialized actors may function as institutional translators, navigating bureaucratic and political interfaces more effectively. This interpretation aligns with policy brokerage theory, which emphasizes the role of boundary-spanning actors who connect political decision-makers, bureaucratic systems, and beneficiary institutions.^8^, ^9^ In higher-magnitude negotiations, such brokerage functions may become particularly salient, as larger funding volumes require coordination across multiple institutional arenas.

The inverse association with extroverted personality profile is particularly noteworthy. While extroversion may facilitate access and networking in lower-magnitude negotiations, higher-magnitude allocation may depend more heavily on strategic planning, technical preparation, and institutional embeddedness rather than interpersonal charisma alone. This finding should be interpreted cautiously, as personality was assessed through a non-validated, single-item perceptual measure and may capture stereotyped role expectations rather than stable psychological traits. These findings challenge simplistic assumptions about relational capital and suggest that allocation scale may demand different behavioral competencies.

Together, these results highlight that discretionary allocation systems are not solely structured by formal rules and partisan alignment but are mediated by the behavioral and positional attributes of individual actors operating within those systems.

### Implications for governance and policy

From a governance perspective, these findings have several implications. First, they underscore the need to acknowledge the human dimension of resource allocation processes in policy analysis. Health systems research has traditionally emphasized institutional design, fiscal architecture, and regulatory frameworks, yet individual-level factors appear to be associated with allocation outcomes.

Second, the centrality of political alignment in higher-magnitude allocation raises normative questions regarding equity and transparency. While political coordination may facilitate larger-scale mobilization, it may also risk reinforcing partisan distribution patterns if not balanced by accountability mechanisms.

Third, the identification of intermediary professionalization as a determinant of allocation scale suggests that institutional capacity-building strategies could influence funding outcomes. Training, formalization of intermediary roles, and clearer procedural criteria may help reduce dependence on informal relational dynamics.

From a public health systems perspective, improving transparency and accountability in discretionary funding processes is critical to ensure equitable distribution of resources. Incorporating structured criteria for intermediary selection and strengthening governance mechanisms may reduce variability driven by informal or relational dynamics, contributing to more efficient and equitable health system performance.

### Strengths and limitations

This study contributes empirically to an underexplored area by integrating individual-level variables into analysis of discretionary public health allocation. The use of multivariable modeling and performance evaluation enhances internal validity.

However, several limitations should be acknowledged. The cross-sectional design precludes causal inference. Because allocation patterns were reported by a single respondent per office, findings reflect perceived usual practices rather than objectively audited financial data. In addition, purposive sampling may limit external validity and introduce participation bias, as offices more engaged in discretionary health funding may have been more likely to participate. Additionally, the study is situated within a specific national discretionary funding context, which may limit external generalizability. Future research should explore longitudinal designs, incorporate administrative datasets, and examine whether similar patterns emerge in other decentralized health systems.

## Conclusion

Parliamentary amendment allocation patterns were associated with political alignment, allocation strategy, and intermediary-level attributes among healthcare resource mobilizers. These findings highlight the relevance of individual-level factors within health resource governance and may inform future strategies to improve transparency and accountability.

## Ethics approval and consent to participate

This study was approved by the Research Ethics Committee of the Faculdade de Medicina da Universidade de São Paulo (CAPPesq), under protocol number 8.151.946 (CAAE 75,513,123.4.0000.0068). The study was conducted in accordance with Brazilian National Health Council Resolution n° 466/2012. Participation was voluntary, and electronic informed consent was obtained from all participants prior to survey completion.

## Consent for publication

Not applicable.

## Declaration of generative AI and AI-assisted technologies in the manuscript preparation process

During the preparation of this work, the authors used ChatGPT (OpenAI) to improve language, readability, and presentation. After using this tool, the authors reviewed and edited the content as needed and take full responsibility for the content of the manuscript.

## Funding

Marcio R. Facanali Jr. received a doctoral scholarship from the Brazilian Coordination for the Improvement of Higher Education Personnel (CAPES), Finance Code 001.

## CRediT authorship contribution statement

**Mariana G. Magon:** Conceptualization, Data curation, Formal analysis, Investigation, Methodology, Visualization, Writing – original draft. **Marcio R. Facanali Junior:** Conceptualization, Methodology, Project administration, Supervision, Writing – original draft, Writing – review & editing. **José Maria Soares Junior:** Methodology, Validation, Supervision, Writing – review & editing. **Sarah Emmely Fazanaro:** Data curation, Investigation, Resources, Writing – review & editing. **Everson L.A. Artifon:** Conceptualization, Methodology, Supervision, Validation, Writing – review & editing.

## Declaration of competing interest

The authors declare no conflicts of interest.

## Data Availability

The datasets generated and/or analyzed during the current study are available from the corresponding author upon reasonable request.
